# Catechol Containing Polyelectrolyte Complex Nanoparticles as Local Drug Delivery System for Bortezomib at Bone Substitute Materials

**DOI:** 10.3390/pharmaceutics12090799

**Published:** 2020-08-24

**Authors:** David Vehlow, Jeremy P. H. Wong, Birgit Urban, Janek Weißpflog, Annett Gebert, Matthias Schumacher, Michael Gelinsky, Manfred Stamm, Martin Müller

**Affiliations:** 1 Leibniz Institute of Polymer Research Dresden, Hohe Straße 6, D-01069 Dresden, Germany; vehlow@ipfdd.de (D.V.); jeremy.wong@epfl.ch (J.P.H.W.); urban@ipfdd.de (B.U.); weisspflog@ipfdd.de (J.W.); stamm@ipfdd.de (M.S.); 2 Department of Chemistry and Food Chemistry, Technische Universität Dresden, Mommsenstaße 4, D-01062 Dresden, Germany; 3 Institute of Bioengineering, École Polytechnique Fédérale de Lausanne, Route Cantonale, CH-1015 Lausanne, Switzerland; 4 Institute for Complex Materials, Leibniz IFW Dresden, Helmholtzstraße 20, D-01069 Dresden, Germany; a.gebert@ifw-dresden.de; 5 Centre for Translational Bone, Joint and Soft Tissue Research, Medical Faculty and University Hospital, Technische Universität Dresden, Fetscherstaße 74, D-01307 Dresden, Germany; m.schumacher@maastrichtuniversity.nl (M.S.); michael.gelinsky@tu-dresden.de (M.G.); 6 MERLN Institute for Technology-Inspired Regenerative Medicine, Maastricht University, Universiteitssingel 40, 6229 ER Maastricht, The Netherlands

**Keywords:** catechol, DOPAC, bortezomib, multiple myeloma, polyelectrolyte complex nanoparticle, drug delivery, controlled release, coating

## Abstract

The proteasome inhibitor bortezomib (BZM) is one of the most potent anti-cancer drugs in the therapy of multiple myeloma. In this study, an adhesive drug delivery system (DDS) for BZM was developed. Therefore, we extended the present DDS concept of polyelectrolyte complex (PEC) nanoparticle (NP) based on electrostatic interactions between charged drug and polyelectrolyte (PEL) to a DDS concept involving covalent bonding between PEL and uncharged drugs. For this purpose, 3,4-dihydroxyphenyl acetic acid (DOPAC) was polymerized via an oxidatively induced coupling reaction. This novel chemo-reactive polyanion PDOPAC is able to temporarily bind boronic acid groups of BZM via its catechol groups, through esterification. PDOPAC was admixed to poly(l-glutamic acid) (PLG) and poly(l-lysine) (PLL) forming a redispersible PEC NP system after centrifugation, which is advantageous for further colloid and BZM loading processing. It was found that the loading capacity (LC) strongly depends on the PDOPAC and catechol content in the PEC NP. Furthermore, the type of loading and the net charge of the PEC NP affect LC and the residual content (RC) after release. Release experiments of PDOPAC/PEC coatings were performed at medically relevant bone substitute materials (calcium phosphate cement and titanium niobium alloy) whereby the DDS worked independently of the surface properties. Additionally, in contrast to electrostatically based drug loading the release behavior of covalently bound, uncharged BZM is independent of the ionic strength (salt content) in the release medium.

## 1. Introduction

Multiple myeloma is a systemic malignant bone disease causing painful bone lesions for patients [1]. The therapy is generally based on two steps. On the one side, these lesions are commonly filled by bone substitute materials (BSM). On the other side, bortezomib (BZM), which is a potent anti-cancer drug [2], is given systemically. Especially, the high therapeutic efficacy of BZM towards myeloma cells in comparison to other malignant cells is well known. In the literature, beside its proteasome inhibition function, which is nonspecific to all eukaryotic cells, BZM also specifically inhibits the NF-κB pathway, which is important for the production of essential growth factors such as interleukin-6 (IL-6) in myeloma cells, is discussed as a specific effect [3,4,5]. However, BZM has side effects. Therefore, BZM therapy using drug delivery systems (DDS) adhesive to bone substitute materials (BSM) could allow controlled, local release of BZM into a bone lesion. Both local as well as retarded proteasome inhibitor release is discussed in the literature as a possibility to overcome drug resistance and the typical relapse of malignant diseases [6]. Recently, we introduced adhesive DDS based on polyelectrolyte complexes (PEC) for local and time controlled delivery of bone therapeutics [7,8]. PEC can be easily prepared by mixing polycation and polyanion solutions, loaded by therapeutic drugs and applied on various model substrates and BSM. Especially, polyelectrolyte pairing between charged polypeptides such as poly(l-lysine) (PLL) and polyglutamic acid used in this work in terms of coacervation behavior and biocompatibility has been well-studied in the literature [9]. Typically, negatively and positively charged drugs such as bisphosphonates and antibiotics can be used, which has advantages due to attractive electrostatic forces between drug and either polycation or polyanion [10]. However, on the one hand, BZM is an uncharged drug, which has disadvantages for DDS based on PEC. On the other hand, it is known that there is specific binding of the boronic acid group of BZM to cis diols, whereby a cyclic diboronic ester group is formed [11,12]. Especially for catechols (i.e., aromatic cis diols) high association constants were found for the boronate ester formation [11]. Several DDS for BZM, based on this reversible and pH-dependent covalent interaction have been already reported [13,14,15]. Therefore, we searched for polycations or polyanions bearing aromatic cis diol groups to exploit the boronic acid esterification in our drug delivery approach of adhesive PEC NP at BSM for local immobilization.

In the literature, PEL based cis diol systems are known based on the oxidative polymerization of catecholamines, where the formation of oligomers or melanin-like polymerisates was claimed [16,17,18]. Recently, we found that oxidative polymerization of catechol containing 3,4-dihydroxyphenylacetic acid (DOPAC) through complex and multiple steps resulted in a polymerisate (PDOPAC) bearing again aromatic cis diols.

The aim of this study was to synthesize the catechol containing polyanion PDOPAC, prepare colloidally stable PDOPAC containing PEC NP, load them with BZM making use of the PEC redispersibility concept [10,19], immobilize them at BSM to obtain adhesive coatings and release the drug in a sustained manner. Thus, a dual strategy to treat multiple myeloma lesions combining a well-established BSM with an effective DDS for the controlled, local delivery of BZM is created. Drug loading and release performance were then mainly characterized by UV/VIS spectroscopy.

## 2. Materials and Methods

### 2.1. Materials

**Figure 1:**
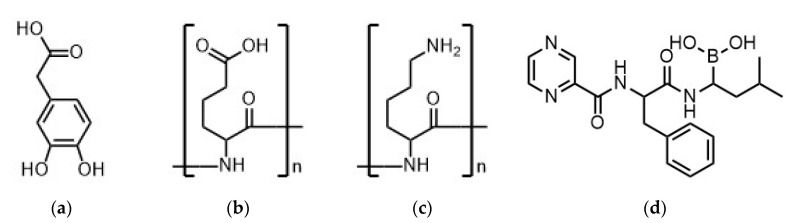
Chemical formulae of (**a**) 3,4-dihydroxyphenylacetic acid (DOPAC); (**b**) poly(l-glutamic acid) (PLG); (**c**) poly(l-lysine) (PLL); and (**d**) bortezomib (BZM).

The monomer 3,4-dihydroxyphenylacetic acid (DOPAC) was purchased from Sigma-Aldrich. As further polyelectrolytes we used the polycation poly(l-lysine) (PLL, 30,000–70,000 g/mol) and the polyanion poly(l-glutamic acid) (PLG, 61,000 g/mol) both from Sigma-Aldrich, St. Louis, MO, USA. The drug bortezomib (BZM) was purchased from Hycultec GmbH, Beutelsbach, Germany. Formulae of polymers and BZM are shown in Figure 1. The HEPES buffer was purchased from Sigma-Aldrich. Solutions were prepared by using Millipore water (water purification system Milli-Q Advantage from Merck Millipore, subsidiary of Merck KGaA, Darmstadt, Germany).

An α-tricalcium phosphate based, hydroxyapatite forming calcium phosphate cement (CPC) precursor mixture was kindly provided from the Gelinsky group (Medical Faculty and University Hospital, TU Dresden, Dresden, Germany) along with the preparation protocol [20]. The unmodified mechanically polished titanium niobium alloy (Ti40Nb) plates were supplied from the Gebert group (Department Chemistry of Functional Materials, IFW Dresden, Dresden, Germany) [21,22]. All used chemicals of this study were at least of p.a. grade.

### 2.2. Synthesis of Catechol Containing PDOPAC

PDOPAC was synthesized via controlled oxidatively induced polymerization under nitrogen atmosphere in a Schlenk flask. In this study, we used only one optimized product with high catechol content for an effective BZM binding. The synthesis of this selected PDOPAC was carried out under the following conditions: 5 mM DOPAC; 10 mM boronic acid; 1 M NaCl; 2.5 mM sodium periodate at pH 8.5 (adjusted with 1 M NaOH), and room temperature. After seven days, the reaction was terminated by filling the reaction mixture in a 50 kDa dialysis membrane (Carl Roth GmbH & Co. KG, Karlsruhe, Germany). Purging was performed for seven days with daily water exchange. Afterwards, the product was freeze dried in a lyophilizer (Free Zone 1, Labconco Corporation, Kansas City, MO, U.S.A.) to obtain a brownish solid powder. Investigations concerning the influence of the reaction conditions such as pH, temperature, and content of oxidizer as well as boronic acid and salt to the product will be published elsewhere.

### 2.3. Preparation of BZM-Loaded Polyelectrolyte Complex (PEC) Nanoparticles (NP)

The drug bortezomib (BZM) was dissolved in a 5.0 mM HEPES buffer at pH 7.4 to a final concentration of 0.25 mM under constant stirring for roughly 24 h. The dialyzed and lyophilized PDOPAC and the two other PEL were dissolved to 2.0 mM in a 5.0 mM HEPES buffer. The complexation of polyelectrolyte complex (PEC) nanoparticles (NP) was performed by mixing defined volumes of PEL without BZM at constant pH 7.4 related to Equation (1). In this study, we adjusted the mixing ratio to n^−^/n^+^ = 0.7 (positive net charge) and to n^−^/n^+^ = 1.4 (negative net charge). The molar charge factor for all polyelectrolytes including the synthesized PDOPAC was determined to be F^+/−^ = 1.00 ± 0.05 as expected for polymers with one charged functional group per repeating unit (see Figure 1).

(1) $$n^{-}{/n}^{+}\;=\;\frac{F_{\mathrm{PA}}^{-}\;\cdot {\;n}_{\mathrm{PA}}^{-}}{F_{\mathrm{PC}}^{+}\;\cdot {\;n}_{\mathrm{PC}}^{+}}$$

$n^{-}{/n}^{+}$ –*molar mixing ratio (net charge)*
$F^{+/-}$ –*molar charge factor of PA (*F*^−^) and PC (*F*^+^)*
$n^{+/-}$ –*molar amount of anionic (*n*^−^) and cationic (*n*^+^) repeating units*

Drug loading was performed by two different techniques: Preloading and postloading. Preloading of PEC NP is related to bulk loading in dispersion. Therefore, the raw particle dispersion (2.0 mL) was centrifuged in Eppendorf tubes for 10 min at 11,000 RPM (Rotana 460 R, Andreas Hettich GmbH & Co. KG). The supernatant was then discarded and the coacervate phase was redispersed in a BZM solution with a defined concentration in a HEPES buffer at pH 7.4. After loading for 16–24 h the particles were again processed. The loading capacity (LC) was determined by spectroscopic analysis (UV/VIS) of the supernatant. After redispersion of the coacervate phase in a 2.0 mL buffer solution the dispersion was again centrifuged. The supernatant was discarded and the coacervate phase was redispersed in 100 μL HEPES buffer to coat BSM. A rinsing step was also performed to eliminate unbound drug. Postloading is referred to drug loading after BSM coating with an original (unloaded) but refined PEC. Therefore, 2.0 mL of PEC NP dispersion was centrifuged and the coacervate phase was redispersed in 100 µL of HEPES buffer to modify BSM. Afterwards, the PEC coated BSM was BZM loaded in 1 cm quartz glass cuvettes by overlaying with 2.0 mL of BZM solution of a defined concentration for 16–24 h. To remove the unbound drug from the surface, the modified BSM was shortly rinsed with pure water. The general processing protocol for purifying and determining the drug loading capacity (LC) of PEC NP dispersions was previously published [10]. In this previous study, charged drugs such as bisphosphonates and antibiotics were loaded instead.

### 2.4. Bone Substitute Materials (BSM)

The general preparation protocol of calcium phosphate cement (CPC) plates was established by Gelinsky group [20]. Pure CPC plates were prepared by mixing 1 g of CPC precursor powder, which contains different calcium phosphate phases and CaCO_3_, with 400 µL of 4% disodium hydrogen phosphate solution carefully. The obtained paste was modelled into molds (diameter of 10 mm and thickness of 1 mm) using a spatula. After self-setting under pressure for 1 day and further hardening for three days under humid conditions, eight CPC plates were obtained. In this study, these plates were used as a microporous BSM in comparison to planar Ti40Nb plates with a smooth surface, provided by the Gebert group [21,22]. The titanium niobium alloy consists of highly pure titanium with 40% niobium. These substrates were modified by solution casting of defined volumes (100 µL) of pure drug solutions or concentrated PEC NP-dispersions with or without BZM depending on the loading technique.

### 2.5. Colloid Titration

Colloid titration was performed using the particle charge detector (PCD-04 (Mütek), BTG, Eclepens, Switzerland) and the corresponding software version 1.00.001 (BTG, Eclepens, Switzerland) to determine the molar charge factors (F^+/−^) of PEL-solutions. This factor F represents the ratio between the true (effective) molar concentration of charged repeating units and that of all repeating units (regardless if charged or not charged) which is necessary to prepare PEC with a defined molar mixing ratio n^−^/n^+^ (see Equation (1)). F = 1 means 100% of (all) the repeating units are effectively charged. F = 0.5 means only 50% of (all) the repeating units are effectively charged. Low molecular 0.001 M PDADMAC or PES-Na with F^+/−^ = 1.000 were used as titrating solutions. The general analyzing protocol was published in a previous manuscript [5].

### 2.6. Quantitative UV/VIS Spectroscopy

Ultraviolet/Visible (UV/VIS) spectroscopy was performed with the spectrophotometer V-650 (Jasco Labor-und Datentechnik GmbH, Groß-Umstadt, Germany) to determine both the drug loading capacities (LC) of PEC NP and the release behaviors of different modified BSM. All measurements were performed in 1 cm UV/VIS quartz glass cuvettes. Drug concentrations were determined by analyzing the intensities of the specific UV/VIS band from BZM at around 270 nm. The spectra were then baseline corrected by dragging the whole line shape to the absorption of 0.000 at 350 nm. A calibration curve was recorded by measuring pure 0.05; 0.10; 0.15; 0.20; 0.25 mM BZM solutions (5.0 mM HEPES, pH 7.4).

Postloading was followed by measuring the drug content in the loading solution at different times to calculate the drug depletion (reverse determination). The loaded BZM concentration was determined by the difference between the initial and the current drug concentration (Equation (2)). Finally, the loading capacity (LC) is defined by Equation (3).

(2) $$c_{\mathrm{BZM}}{(\mathrm{loaded})\;=\;c}_{\mathrm{BZM}}{(\mathrm{initial})\;-\;c}_{\mathrm{BZM}}\left(\mathrm{current}\right)$$

(3) $$\mathrm{LC}\;\left[\%\right]=\;\frac{c_{\mathrm{BZM}}\left(\mathrm{loaded}\right)}{c_{\mathrm{BZM}}\left(\mathrm{initial}\right)}\times \;100\%\;=\frac{c_{\mathrm{BZM}}{(\mathrm{initial})\;-\;c}_{\mathrm{BZM}}\left(\mathrm{current}\right)}{c_{\mathrm{BZM}}\left(\mathrm{initial}\right)}\times \;100\%$$

$c_{\mathrm{BZM}}\left(\mathrm{loaded}\right)$–*concentration of adsorbed BZM in PEC NP*
$c_{\mathrm{BZM}}\left(\mathrm{initial}\right)$–*concentration of BZM before loading*
$c_{\mathrm{BZM}}\left(\mathrm{current}\right)$–*concentration of BZM after loading (supernatant)*

Drug loading of preloaded PEC NP was quantified by measuring the BZM content in the supernatant after centrifugation as described in Section 2.3. In addition to the already mentioned baseline correction a further spectrum manipulation step was necessary to correctly determine the absorption at 270 nm and out of that the c_BZM_(loaded) as well as LC. The supernatant after preloading contained a small but not negligible content of PDOPAC/PEC which showed also absorptions in the relevant spectral range. Consequently, the UV/VIS spectrum of the pure PDOPAC/PEC had to be subtracted in a defined manner. This was done by the minimization of the standard deviation using the “target value analysis” tool in Microsoft Office Excel for all wavelengths in the range of 250–300 nm as shown in Equation (4).

(4) $${s(c}_{\mathrm{BZM}})\;=\overset{300}{\underset{\lambda =250}{\sum }}(\frac{A_{\lambda }}{\varepsilon_{\lambda }}-\bar{\frac{A_{\lambda }}{\varepsilon_{\lambda }}})$$

${s(c}_{\mathrm{BZM}})$ –*standard deviation of BZM concentration (calculated out of all calibration lines in the wavelength range between 250–300 nm) [mM]*
$A_{\lambda }$ –*absorption at a defined wavelength [-]*
$\bar{A_{\lambda }}$ –*average of all absorptions of all wavelength (250–300 nm) [-]*
$\varepsilon_{\lambda }$ –*absorption coefficient for a defined wavelength [1/mM]*
$\bar{\varepsilon_{\lambda }}$ –*average of all absorption coefficients of all wavelength (250–300 nm) [1/mM]*

**Figure 2:**
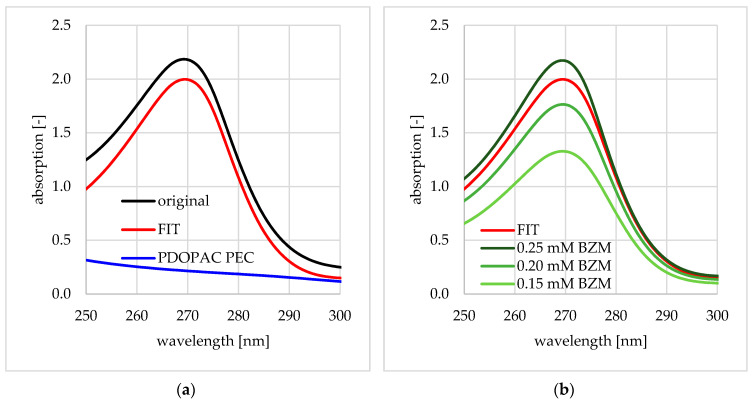
Well-defined subtraction of UV/VIS spectra related to Equation (4). In (**a**) the UV/VIS spectra of BZM containing the supernatant before and after subtraction as well as the pure PDOPAC/PEC spectrum (subtrahend) and in (**b**) the corrected spectrum of the supernatant in comparison to pure BZM spectra of selected concentrations is shown.

Via this method, it was possible to remove the PDOPAC/PEC signals out of the original (sum) spectrum to create a spectrum representative to BZM of the unknown concentration, which is shown in Figure 2. Finally, the loading was calculated as defined in Equations (2) and (3).

To measure drug release, differently modified Ti40Nb and CPC plates were incubated with 2 mL of release medium (Millipore water) in 1 cm UV/VIS quartz glass cuvettes. Time dependent drug enrichment (direct determination) in the release medium was monitored. Respective BZM concentrations were calculated using calibration curves. Finally, drug concentrations of each specific moment in time (c_BZM_ (current)) were compared with the loaded drug content (c_BZM_ (loaded)) of the sample to calculate the released BZM amount (see Equation (5)). The drug release was given in BZM percentage (BZM [%]) versus time. The residual content (RC) of drug (BZM) in the coating is defined by Equation (6).

(5) $$\mathrm{BZM}\;\left[\%\right]\;=\;\frac{c_{\mathrm{BZM}}\left(\mathrm{current}\right)}{c_{\mathrm{BZM}}\left(\mathrm{loaded}\right)}\times \;100\%$$

RC [%] = 100% − BZM [%] (at t)(6)

Measured data were fitted as described in our previous work [23] related to protein release characterization via Ritger/Korsmeyer/Peppas analysis related to Equation (7) [24,25]. The obtained release parameters A_0_ and B characterize the type of release, whereby A_0_ represents more or less the initial burst behavior and B the type of diffusion.

A(t) = A_0_⋅t^B^ or A(t) = 100 − A_0_⋅t^B^ (7)

A(t) –*drug content [%/mM]*
T –*time [h]*
A_0_ –*amplitude [%/mM]*
B –*exponent [-]*

### 2.7. Quantitative FTIR Spectroscopy

Fourier transform infrared (FTIR) spectroscopy was performed using the Vertex V70 spectrometer (Bruker Optics GmbH, Ettlingen, Germany) controlled by the OPUS software (Bruker, Ettlingen, Germany) in the attenuated total reflection (ATR) mode using a single beam 4-mirror-ATR attachment (Perkin Elmer GmbH, Überlingen, Germany). First, a reference intensity spectrum I_R_ of the uncoated germanium internal reflection element (Ge IRE) was recorded. After solution casting, drying in the oven at 50 °C and further flushing in a gentle N_2_-stream, the coated Ge IRE was measured to obtain the sample intensity spectra I_S_. I_S_ and I_R_ spectra were related to an absorption spectrum according to A = −log (I_S_/I_R_). Each intensity spectrum was measured with a spectral resolution of 2 cm^−1^ and 100 scans were averaged. To measure time dependent release kinetics, the DDS coated Ge IRE was placed in an in situ cell equipped with a liquid chamber filled with various release media. After defined times the media were removed. The liquid chamber was then rinsed with fresh Millipore water and dried in a gentle N_2_-stream. Measured data were analyzed as described above (Section 2.6) using Equations (5)–(7). The absorption of interest is around 1020 cm^−1^, assigned to an IR active vibration of BZM that shows no overlapping with other components. Thus, we analyzed the entire absorption values of this band after cutting between 1090–1010 cm^−1^ and then performed baseline correction. Furthermore, IR spectroscopy was performed in transmission mode to characterize the adhesivity of PEC coatings at Ge model substrates.

### 2.8. Inductively Coupled Plasma Optical Emission Spectroscopy (ICP-OES)

Inductively coupled plasma optical emission spectroscopy (ICP-OES) was used to determine the boron or BZM concentration of selected samples to confirm the results of UV/VIS spectroscopy. Measurements were performed with the ICP-OES spectrometer iCAP 7400 from Thermo Scientific (Dreieich, Germany) with the software Qtegra Intelligent Scientific Data Solution. Each investigated sample was measured in triplicates. The calibration curve was constructed using four calibration standards: 0, 3.125, 6.25, 12.5, and 25 mg L^−1^ of boron (S4400-5M74) purchased from CPI international (Amsterdam, Netherlands). Data analysis was then performed for the carefully selected wavelength of boron at 182.591 nm.

### 2.9. Dynamic Light Scattering (DLS)

Dynamic light scattering (DLS) was applied to determine the hydrodynamic radius R_H_ of PEC-NP in dispersion based on the translational diffusion coefficient D using the Stokes-Einstein Equation R_H_ = k_B_T/(6Dπη) with Boltzmann constant k_B_, temperature T (25 °C), and viscosity η. Measurements were performed with the Jianke Portable Particle Sizer from Jianke Instruments Co. Ltd. (Wuhu, China) at a constant scattering angle of 89.3° using 2 mL samples in cylindrical glass cuvettes with a diameter of 10 mm. The autocorrelation function (ACF) was recorded for 180 s. For the calculation of the particle sizes and polydispersity indices (PDI) based on ACF the ALV-5000/E/EPP-Software of ALV GmbH (Langen, Germany) was used applying the tools “Regular Fit” and “Simple Fit”.

### 2.10. Zeta Potential (ZP)

Zeta potential (ZP) measurements of PEC NP were performed at the ZetaSizer Nano ZS (Malvern Instruments Ltd., Worcestershire, UK). U-shaped tube cuvettes were used, in which the PEC NP dispersion at pH = 7.4 was carefully filled avoiding air bubbles. In the dedicated Zetasizer software (version 7.13) the voltage was set to 40.0 V and as material standard (reference) “polystyrene latex” was chosen. For each ZP measurement, 50 single measurements were performed in triplicate at T = 25 °C and the average value was taken with the respective standard deviation.

### 2.11. Scanning Force Microscopy (SFM)

Microscopic appearance of PEC NP coatings at substrates was analyzed by scanning force microscopy (SFM). Measurements were performed in non-contact-mode with a constant scanning speed of 16.0 μm s^−1^ at the Nanostation II and the attached software SIScan Panel version 1.7 g from Bruker Nano GmbH (Karlsruhe, Germany) was used. Silicon nitride probe tips from Nanosensors (Darmstadt, Germany) with an apex of around 10 nm were used. Topography mode SFM images were post processed using SPIP (software package for nano- and microscale image processing) from Image Metrology (Horsholm, Denmark).

## 3. Results

**Figure 3:**
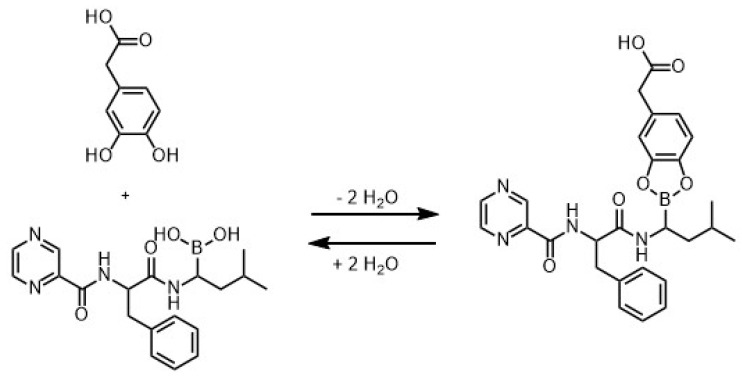
Reversible covalent interaction (esterification) between the drug BZM and catechol containing DOPAC.

First, the selected PDOPAC product was qualitatively checked for catechol amount. Related ATR-FTIR spectra of DOPAC and its polymerizate PDOPAC were recorded, which are given in Figure S1. Significantly, the absorption at around 1260 cm^−1^ for DOPAC assigned to phenols is (partly) preserved in PDOPAC. Furthermore, the reversible covalent binding (esterification) of BZM to our synthesized catechol bearing polyelectrolyte is shown for the monomer DOPAC in Figure 3. Figure S2 shows ATR-FTIR spectra of PDOPAC before and after BZM interaction. Significantly, at 1080 cm^−1^ a small IR band appears, which could be assigned to the cyclic ester between boronic acid and cis diols reported in [12] as one of the specific vibrations of the conjugate.

### 3.1. Colloid and Adhesive Properties of PDOPAC/PEC NP

**Figure 4:**
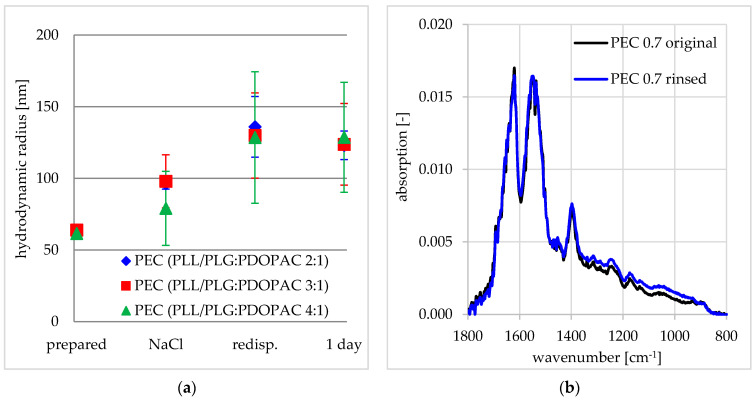
Colloid and adhesive properties of PDOPAC/PEC 0.7. (**a**) Evolution of hydrodynamic radii of the nanoparticles in dispersion measured by dynamic light scattering (DLS) and (**b**) transmission IR spectra of PEC coatings before and after rinsing.

In Figure 4a, DLS data on the herein used ternary PEC NP of poly(l-lysine)/poly(l-glutamic acid)/PDOPAC (PLL/PLG/PDOPAC) with different PLG/PDOPAC ratios for various colloid processing states are provided. Obviously, the ternary PEC particles have similar hydrodynamic radii for each of the processing states and thus PLG/PDOPAC ratios have no significant effect on PEC particle size. However, the size of the particles increased upon salt addition as well as upon redispersion upon centrifugation (see Section 2.3). The hydrodynamic radius of the particles remained constant upon storing the PEC samples for 24 h, suggesting relatively high colloidal stability. Moreover, the count rates and the polydispersity indices did not change significantly (see Figures S3 and S4). The anionic PDOPAC/PEC 1.4 shows a similar behavior (see Figures S5–S7). The maximal ratio of PLG/PDOPAC (polyanions) was found to be 3:1 in cationic and 4:1 in anionic PEC to retain the beneficial property of redispersibilty upon centrifugation of the PEC NP dispersion. Long-term colloid stability and especially the possibility of multiple processing steps were reached.

Zeta potential measurements at PDOPAC/PEC 0.7 and PDOPAC/PEC 1.4 at pH = 7.4 (HEPES buffer) revealed a positive net charge of 29.4 mV and a negative one of −32.0 mV, respectively, which is summarized in Table S2 of SM. Obviously, PEL bound excess charge of these PEC NP has equal magnitude regardless of charge sign. Hence, excess charge is assumed to be located rather in the shell of PEC NP, which prevents PEC NP from aggregation due to electrostatic repulsion. This behavior is necessary for the storage of dispersions and the drug preloading concept described later.

Furthermore, the adhesive stability at the relevant BSM is important for the loading and release studies as well as the planned application, which can be characterized by FTIR spectroscopy. From Figure 4b, the wet adhesiveness for centrifuged ternary PDOPAC/PEC 0.7 can be rationalized. Notably, absorption within the transmission FTIR spectrum for the PEC coating before and after rinse does not decrease. Instability in terms of adhesivity of the PEC coating would lead to significant loss of either the absorption bands of polycation or polyanion, which has been shown for uncentrifuged PLL/cellulose sulfate PEC NP films in the range between n^−^/n^+^ 0.5–1.5 [10].

**Figure 5:**
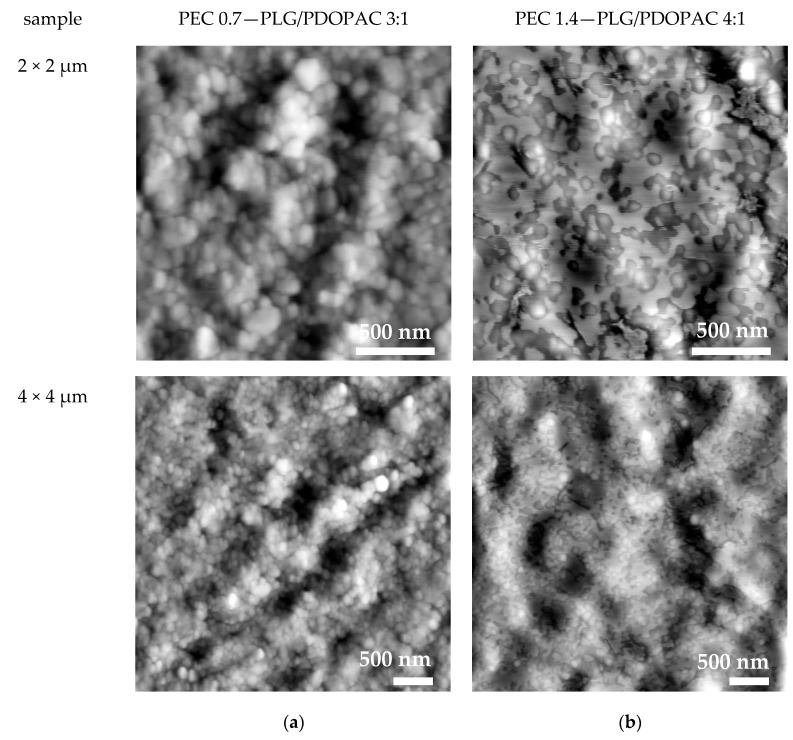
SFM images of PEC coatings from (**a**) cationic and (**b**) anionic ternary PDOPAC/PEC after centrifugation and redispersion under different magnifications.

These highly wet-adhesive PEC/PDOPAC coatings were further studied by SFM. From Figure 5, a significant difference in the appearance of films of PEC NP with various n^−^/n^+^ can be obtained. However, single particles are not recognizable in the PEC 1.4 coating. Obviously, ternary anionic PEC NPs were merged. The reason for particle fusion could be a less stable shell due to the use of a polyanion mixture (with different macromolecular weights). Thus, it is not possible to determine particle sizes of anionic PEC in the solid state by SFM, which however might be estimated from DLS. In contrast, isolated single NPs could be observed for cationic PEC 0.7 coatings. However, particle size distribution was rather large. Comparing SFM data on PEC 0.7 NP coatings and DLS data on respective dispersions, smaller particle sizes were found for the PEC coatings, which could potentially be explained by the shrinking of particles due to water loss.

### 3.2. Loading of BZM into PDOPAC/PEC NP Dispersions and Coatings

As described in Section 2.6, the drug content in the loading and release media was determined mainly by UV/VIS spectroscopy. Further, ATR-FTIR and ICP measurements were performed to confirm the UV/VIS quantified drug loading and release amounts, respectively. We investigated two loading techniques as described in Section 2.3 and compared the results between the uncharged BZM with other charged drugs (bisphosphonates) of our former studies [10].

#### 3.2.1. Preloading into PDOPAC/PEC Dispersions

##### Influence of Ionic Strength

**Figure 6:**
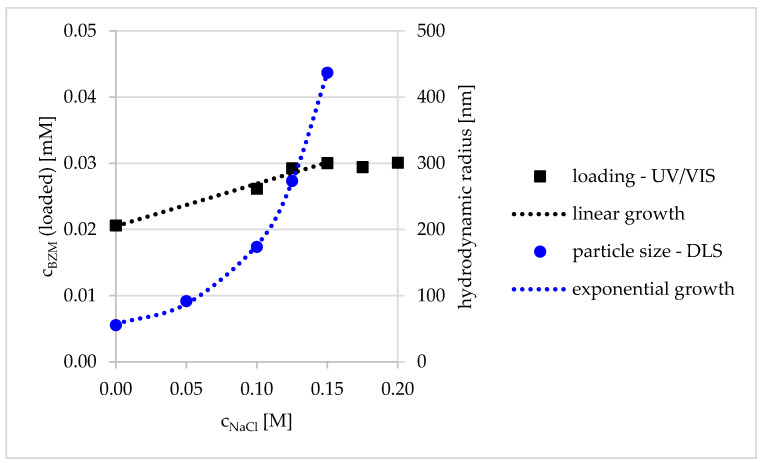
Linear increase of loading capacity and exponential particle growth of PDOPAC/PEC with rising salt concentration from 0–0.15 M NaCl in the dispersion.

It was not possible to coacervate the complete PEC dispersion (i.e., locally densified and phase separated) via centrifugation and redispersed via shaking without any additives, which was also visually observable. Therefore, to overcome this problem, a defined amount of salt was added into the PEC dispersions to induce particle growth in a defined manner. The effect of salt on particle size of PEC NP is well known and was reported, e.g., for PDADMAC/PSS complexes [26]. By increasing the concentration of sodium chloride, the LC shows a linear increase, whereas the particle size was growing exponentially (see Figure 6). At concentrations of NaCl above 0.15 M, no further increase in LC was reached. Moreover, the complete coacervative deposition was visually observable. Presumably, the increasing LC with salinity is a sub effect of the exponential particle growth, which is empirically described in Equation (8). The increase in particle size with small amounts of added salt arises from swelling of PEC NP, which was studied in detail by Tirrell [27]. Based on the same reference, disassembly of PEC aggregates at extremely higher NaCl concentrations would be expected which would probably lead to decrease in LC. However, the salt content does not affect the BZM–catechol interaction. In further experiments, we adjusted the salt concentration to 0.1 M. We recognize this value as an optimum between particle size and LC (PEC NP deposition during centrifugation). Furthermore, the LC is approved by an additional method ICP-OES for the selected sample (0.1 M NaCl). The loaded BZM concentration of 0.026 ± 0.004 mM is in good agreement with c_BZM_ (loaded) = 0.024 ± 0.002 mM obtained by UV/VIS.

(8) $$R_{H}\;=\;42.10\;+\;15.55\;\times \;e^{{(c}_{\mathrm{NaCl}}/0.04638)\;}\;\mathrm{for}\;0\;<\;c_{\mathrm{NaCl}}\;<\;0.15\;\mathrm{in}\;[M].$$

##### Influence of PDOPAC Amount

**Figure 7:**
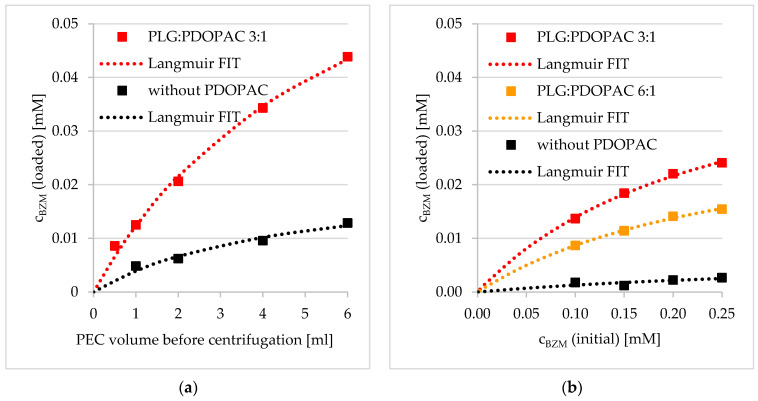
Loading capacity of PDOPAC/PEC in dependence of (**a**) volume of the dispersion and (**b**) initial BZM concentration for two different polyanion ratios (preloading).

The influence of the PDOPAC amount on LC in PEC NP was investigated in two different ways. As shown in Figure 7, we either increased the entire PEC amount (in 2 mL) via changing the concentration of the PEC dispersion (Figure 7a) or we varied the PLG to PDOPAC ratio in the particles itself (Figure 7b). In both cases, an increase of the c_BZM_ (loaded) with rising catechol content in the system was observable. In comparison to the reference PEC, the LC is directly dependent on the PDOPAC concentration in the dispersion. These results suggest that BZM binds covalently to the catechols in PDOPAC and also that the PDOPAC contains catechols in a sufficient amount to interact with the drug. The differences of the PLG:PDOPAC 3:1 sample (2 mL/0.25 mM BZM) between the two diagrams related to the LC result from the above described effect of salt. In Figure 7, the PDOPAC/PEC sample with 0.1 M NaCl before centrifugation exhibits a higher c_BZM_ (loaded). However, the LC without salt is a little bit higher than in the 0.1 M NaCl sample if comparing the PEC without PDOPAC (black squares at 2 mL in Figure 7a and at 0.25 mM BZM in Figure 7b). Obviously, the physisorption of PEC NP in the medium with a very low ionic strength is more effective. However, in both cases (independent of the salt content) the reference PEC without PDOPAC shows a significantly lower BZM loading.

Furthermore, it is possible to fit both, the data concerning BZM loading versus PEC volume and that versus initial c_BZM_ with a Langmuir isotherm. This adsorption model is based on monolayer adsorption, which does not really apply for BZM bound at soft and porous PEC NP in dispersion. Presumably, the good agreement with the Langmuir model is rather based on the available chemisorption sites described by the amount of functional groups, which are able to bind the drug specifically. In this sense, the monolayer covering (degree of covering = 1) means the saturation of the entire catechol groups in the PDOPAC/PEC dispersion by BZM. The Langmuir isotherm, which was used to fit BZM loading data, is defined by Equation (9). Related fit parameters c_max_ and K_L_ including accuracy (R^2^) are given in Table S1. Interestingly, c_max_ correlates with the PDOPAC amount in the PEC NP.

(9) $$c_{\max}\;=\frac{K_{L}\;\times \;x}{{1\;+\;K}_{L}\times \;x}\;\mathrm{whereby}\;x\;=\;c_{\mathrm{BZM}}\;(\mathrm{initial})\;\mathrm{or}\;\mathrm{PEC}\;\mathrm{volume}\;\mathrm{before}\;\mathrm{centrifugation}$$

##### Influence of Net Charge

**Figure 8:**
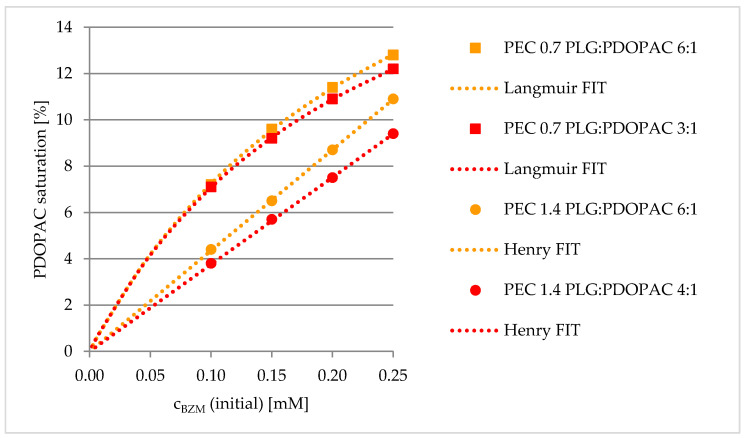
Effect of net charge (PEC 0.7, PEC 1.4) and PLG/PDOPAC ratio on PDOPAC saturation in dependence of initial BZM concentration.

The effect of PEC NP net charge on LC was studied by analyzing the specifically bound amount of BZM to the catechols of PDOPAC. Under PDOPAC saturation the true PDOPAC amount in the dispersion (incomplete separation with 0.1 M NaCl) and the physisorption (BZM binding to PEC without PDOPAC) was considered (see Figure S8). Furthermore, it is beneficial to compare these PDOPAC referred loadings because of varying amounts of PDOPAC in the cationic and anionic PEC NP with the same PLG/PDOPAC ratio. In Figure 8, it is shown that the positively charged PEC 0.7 has a higher affinity to the drug independent of the polyanion ratio. To explain this phenomenon we referred back to the chemical structural organization of the PEC NP. In the case of cationic PEC, the PDOPAC as one of the polyanions is mostly located in the core of the particles. Consequently, the catechols, which are essential for the drug binding, are mainly located in the inner part of the NP, protected by the excess polycation dominated shell. In contrast, for the anionic PEC 1.4, PDOPAC is evenly distributed across the whole particle, especially in the polyanion dominated shell. We further assume that the water content in the inner regions of the particle (core) is smaller than in or at the shell. Since the esterification reaction (see Figure 3) is promoted, when the reaction product water is rare, the BZM–catechol interaction will be stronger in the dehydrated core of the PEC NP. Hence, the effect of PEC NP net charge on the loading is assumed to be due to different PDOPAC distributions for PEC 0.7 compared to PEC 1.4. Furthermore, the type of isotherm is influenced by the PEC net charge. While for PEC 0.7 (cationic excess charge), the Langmuir model represents again well the concentration dependent PDOPAC saturation (loading), PEC 1.4 (anionic excess charge) shows a linear dependency. This might be attributed to the initial Henry regime of a Langmuir isotherm being valid for extremely low drug or high PDOPAC concentrations, respectively. Obviously, for PEC 1.4 the investigated BZM concentration (c_BZM_ before loading) range is too small. Presumably, the maximum possible LC is larger for PEC 1.4 than for PEC 0.7, because the accessibility of catechols is much better due to the high PDOPAC concentration in the shell. However, the higher water content in the shell in comparison to the core, has a stronger effect and leads to the lower drug chemisorption by the esterification reaction as described above.

#### 3.2.2. Postloading of PDOPAC/PEC Coatings at Ti40Nb

**Figure 9:**
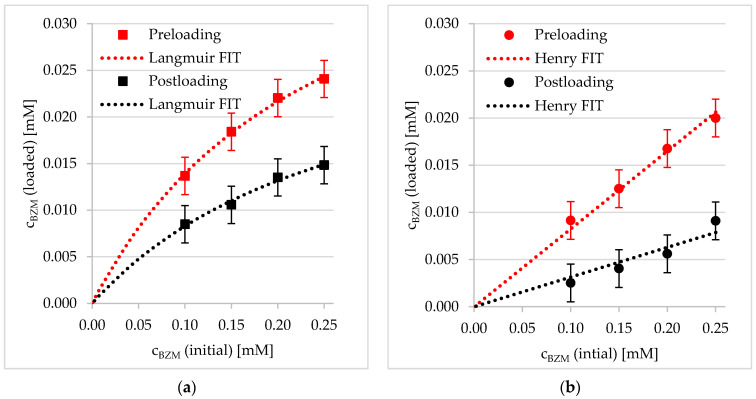
Comparison of adsorption isotherms of pre- and postloaded PEC NP for (**a**) PEC 0.7 with positive net charge and (**b**) PEC 1.4 with negative net charge.

Drug loading of PDOPAC/PEC coatings at Ti40Nb was studied in dependence of the net charge of the PEC NP (PEC 0.7 versus PEC 1.4). Analogous to preloading, a different sorption behavior of BZM at PEC NP coatings with cationic compared to anionic net charge was observable for postloading, which is shown in Figure 9. In the case of postloaded PEC NP coatings, the absolute values of c_BZM_ (loaded) are significantly lower for both positive as well as negative net (excess) charge. Presumably, less catechols are available for BZM interaction in the coatings compared to the dispersions, which might be explained by fewer and smaller diffusion paths in the film formed by particle shrinking during drying. Nevertheless, for both loading techniques, the types of the isotherm regimes are similar for the respective PEC 0.7 (Langmuir) and PEC 1.4 (Henry) coating. This obvious difference in the adsorption process at PEC 0.7 compared to PEC 1.4 seems to be rather dependent on the PDOPAC distribution (see above) and independent on size (no significant differences in hydrodynamic radii (100 nm < r_h_ < 200 nm) in dependence of PEC NP net charge). Presumably, the decrease of loading (c_BZM_ (loaded)) due to the reduced catechol availability is not only based on PEC NP shrinking itself, but also on formation of a dense PEC NP layer (agglomeration), resulting in reduced water permeability. This assumption is supported by the SFM images on PEC NP coatings of Figure 5 showing fused particles for PEC 1.4.

#### 3.2.3. Kinetics of Drug Loading

**Figure 10:**
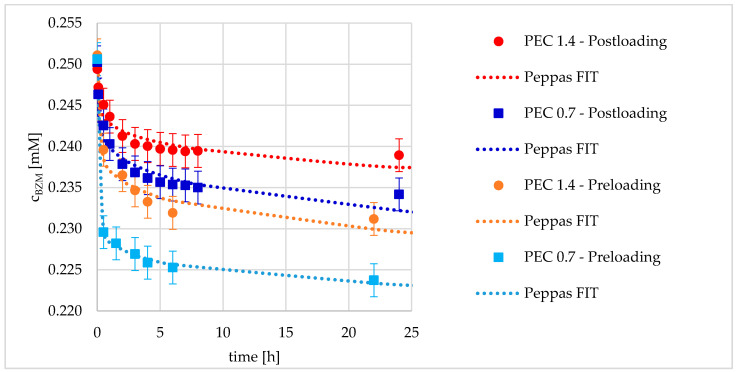
Kinetics of BZM loading in dependence of the net charge and type of loading.

In addition to the concentration dependence (c_BZM_) of the loadings measured after an equilibration time of 16–24 h, the time dependence of the loadings at a fixed initial c_BZM_, i.e., the sorption kinetics was investigated. The results of the sorption kinetics for PEC 0.7 and PEC 1.4 under preloading and postloading conditions, respectively are provided in Figure 10 (note that c_BZM_ is related to the depleted BZM amount in solution and not to loaded amount). The time dependent trends for these sample combinations were found to be similar to the concentration dependent trends shown in Figure 9. BZM loading was higher for preloading compared to postloading and BZM loading was found to be highest for PEC 0.7. Furthermore, the initial burst uptake dominates the loading process, with the result that the equilibrium concentration is nearly reached after roughly 5 h. Furthermore, the model of Ritger, Korsmeyer, and Peppas is well suitable for the representation of the time-dependent loading data (at least at smaller times). This empirical power law was originally developed for the description of drug release out of a polymer matrix. Obviously, it is also suitable for the representation of drug loading. Hence, it could be concluded that the loading process is dominated by drug diffusion into PEC NP, and the rate-determining step is not the esterification (see Figure 3) but rather diffusion associated drug adsorption.

To discuss the differences between the samples, kinetic parameters of the fitting model are shown in Table 1. The amplitude A_0_ is a parameter related to the initial uptake amplitude and exponent B describes the type of uptake kinetics being Fickian diffusion for B = 0.5. The diffusion into the PEC NP in dispersion is less hindered in comparison to coatings, which is shown by the significantly lower exponent B for preloaded PEC NP for both net charges (see Table 1). The minor availability of the catechol groups in the coatings leads to the smaller loading capacity, which was already described in the previous sections. Herein, the respectively lower amplitude A_0_ for postloading in comparison to preloading confirms this assumption.

#### 3.2.4. Comparison to Charged Drugs

**Figure 11:**
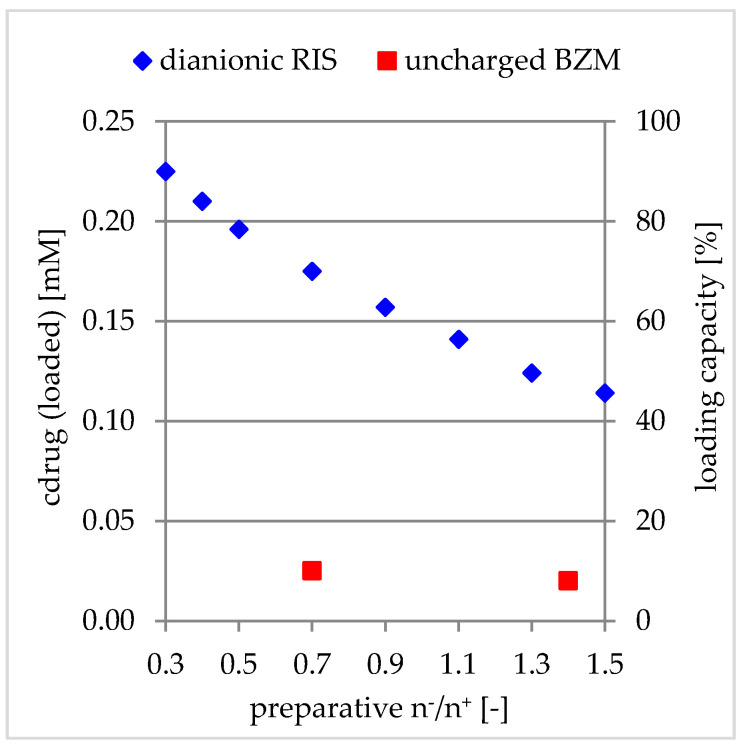
Loading capacity of PEC NP in dependence of net charge related to uncharged BZM (PLL/PLG/PDOPAC) (red) in comparison to dianionic risedronate (PLL/CS1.0) (blue).

In our previous work, the loading capacity of PEC NP coatings towards the dianionic risedronate (RIS) in dependence on the net charge of PEC (PEC 0.5 to 2.0, i.e., n^−^/n^+^ = 0.5–2.0) was investigated [10]. A more or less linear increase with rising cationic charge was found based on electrostatic interactions between the negatively charged drug and the polycation. In Figure 11, we compared these loading capacities with the determined ones for the uncharged BZM. Significantly, the absolute c_drug_ (loaded) values are lower for the covalent compared to electrostatic binding. This is most likely due to low PDOPAC amounts and thus, less reactive catechol sites in the PEC NP compared to high numbers of charged PEL sites. By increasing the PDOPAC- and thus the catechol- content, no matter in which way (see Figure 7), the LC rises. Furthermore, the influence of the net charge of PEC NP on LC of BZM based on the PDOPAC distribution in the particles, described in Section 3.2.1, is negligible compared to that for the charged drugs. The most important factor for drug loading based on covalent binding is the availability of specific functional groups (catechols for esterification). In contrast, for the more or less unspecific electrostatic interaction the excess of polycation or polyanion in the PEC NP is crucial for the effective drug loading.

### 3.3. Release of BZM from PDOPAC/PEC Coatings

**Figure 12:**
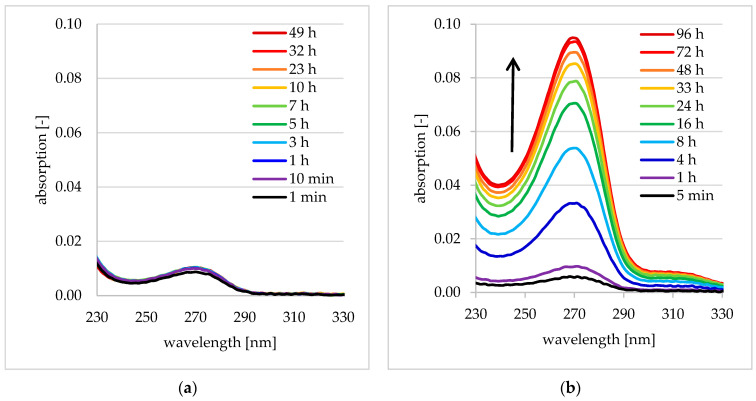
UV/VIS spectra of release media during the release of BZM from (**a**) binary PLL/PLG PEC and (**b**) ternary PDOPAC/PEC coating at Ti40Nb.

In addition to the drug loading described in Section 3.2, the drug release behavior is also a crucial property for determining the performance of the DDS. In Figure 12, UV/VIS spectra recorded during the incubation of BZM loaded binary PEC (PLL/PLG) and ternary PDOPAC/PEC (PLL/PLG/PDOPAC) coatings with release medium are shown. While the BZM loaded binary PEC without PDOPAC (Figure 12a) did not show any sustained retarded release of BZM, a long term retarded BZM release was obtained for the BZM loaded ternary PEC with PDOPAC, which can be rationalized from the course of the drug assigned signal at 270 nm shown in Figure 12b. Hence, the chemically caused retention of BZM at complexed PDOPAC is obvious.

As was already outlined for the loading properties, the covalent binding between BZM and catechols is based on esterification, which is a condensation reaction with ester groups and water molecules as product. According to the law of mass action this reaction is reversible. Hence, locally adding water by influx into the BZM loaded PEC NP coating stimulates the hydrolysis of the BZM-PDOPAC ester and free BZM molecules as former educts are able to diffuse through the PDOPAC/PEC layer.

#### 3.3.1. Release Performance from Germanium Model Substrates

**Figure 13:**
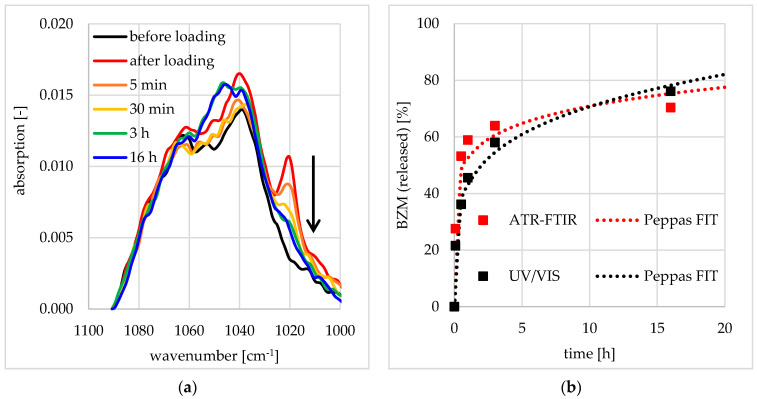
ATR-FTIR spectra on the release of BZM from PEC nanoparticles (NP) coatings deposited at the germanium model substrate (**a**) and the release kinetics found on ATR-FTIR in comparison to UV/VIS data (**b**).

In order to determine the BZM release kinetics of the pure DDS without any surface effects at PDOPAC/PEC coatings, planar germanium crystals and ATR-FTIR spectroscopy were chosen as complementary model substrates and analytical technique, respectively. The results are provided in Figure 13, where the ATR-FTIR spectrum recorded after loading clearly shows drug enrichment into the PDOPAC/PEC layer upon the increase of the band at 1020 cm^−1^ assigned to BZM. During the release this specific absorption decreases in a continuous manner. The enrichment of the BZM in the release medium is simultaneously measured by UV/VIS spectroscopy. The drug release kinetics determined by both methods are shown in Figure 13b. Similar time dependent courses and values were observed for ATR-FTIR and UV/VIS detection, confirming complementary results. Furthermore, the kinetics can be described by a diffusion of a drug through a polymer matrix according to the power law of Ritger, Korsmeyer, and Peppas given in Equation (7) for both methods. Since the UV/VIS data show a lower deviation between experimental and fit data, this method was chosen for the further characterization and quantification of BZM release. The release performance of this postloaded sample at the germanium model substrate will be discussed later on.

#### 3.3.2. Release Performance at Bone Substitute Materials (BSM)

The influence of the varied parameters during loading, described in Section 3.2 and additionally, the effect of the substrate will be investigated for the release behavior of PEC coatings at medically relevant BSM. Therefore, pre- and postloaded PEC NP coatings at Ti40Nb and CPC were incubated in a release medium.

##### Influence of Substrate

**Figure 14:**
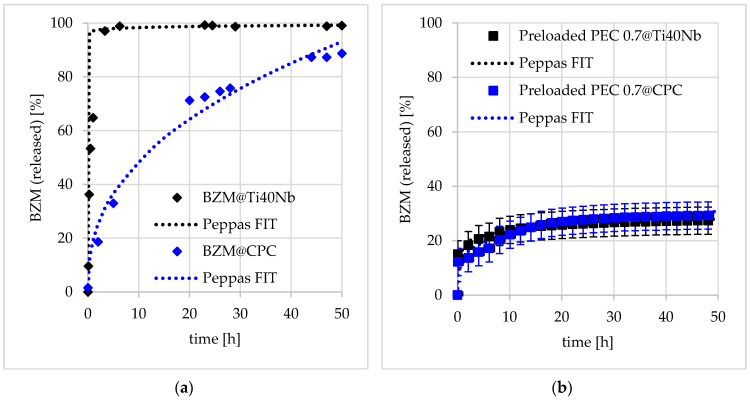
Release kinetics of (**a**) pure BZM coating and (**b**) BZM loaded PDOPAC/PEC coating at Ti40Nb as well as CPC (calcium phosphate cement).

In order to evaluate the influence of the substrate on the release behavior, the relevant bone substitute materials (BSM) CPC and Ti40Nb were functionalized by both the pure drug and the drug loaded PDOPAC/PEC and the release kinetics upon contact to the aqueous medium was determined. The release performance based on UV/VIS measurements is shown in Figure 14. The pure BZM layer at Ti40Nb is washed away spontaneously and completely. Obviously, the BZM without DDS is not adhesive at planar substrates. A certain retardation was reached for pure BZM coated onto CPC. Probably, the BZM does not form a surface layer but rather penetrate into the microporous bulk material. Hence, the drug is drying in the cavities of the porous material and the following rinsing results in a hindered diffusion process. Thus, the unmodified bone cement represents already a DDS for the investigated drug. In Figure 14b, the release kinetic of preloaded PEC NP coatings at both Ti40Nb and CPC are shown. In comparison to the pure BZM modified substrates, a strong retardation of the drug using the PDOPAC/PEC is observable for both BSM. Hence, the effect of the substrate is more or less invariant. There exists some little kinetic differences, but given the error bars these are of no significance. Obviously, in contrast to the pure drug the drug loaded PEC particles did not penetrate into the porous bulk material of the CPC and thus, the retardation of the PEC NP layers at both materials are comparable. Hence, we conclude, that PDOPAC/PEC NP coatings function uniquely at various BSM, even if they are as different as CPC and Ti40Nb.

Furthermore, the released BZM amount of the preloaded PDOPAC/PEC 0.7 found by UV/VIS was proofed by ICP-OES measurements. Both methods showed similar values of relative BZM released amount after 48 h, which was roughly 30% for the investigated cationic PEC at Ti40Nb.

##### Influence of Net Charge and Type of Loading

**Figure 15:**
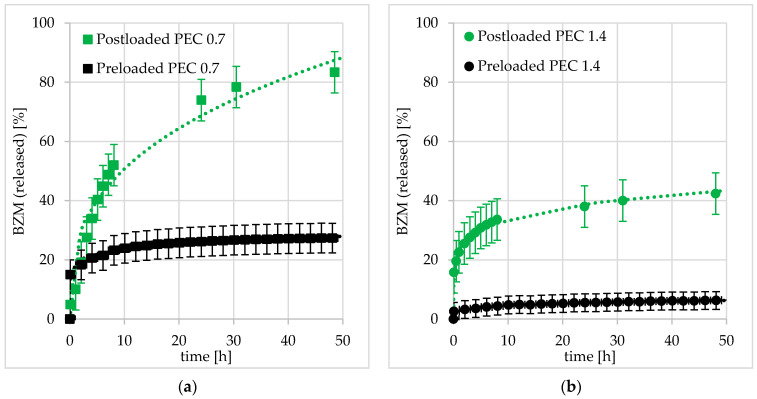
Release kinetics of BZM from (**a**) positively charged PEC 0.7 and (**b**) negatively charged PEC 1.4 in dependence of loading type.

In Section 3.2, it was concluded that both, net charge having a significant effect on the colloid structure of PDOPAC/PEC NP and especially the type of loading (pre- or postloading) are crucial parameters for the loading of BZM. In the following, the influence of both parameters on the release performance is investigated. The results obtained are shown in Figure 15a for cationic PEC 0.7 and 15b for anionic PEC 1.4.

The received release kinetics were quite different. Postloaded PEC NP systems always displayed a high and long-term (sustained) release compared to preloaded ones. Especially for cationic PEC 0.7, where postloading (see Figure 15a) leads to an almost complete BZM release within two days. Obviously, the entire diffusion paths which were used during postloading are available for the drug release. In Section 3.2.2, the decreased LC of postloaded PEC in comparison to preloaded ones was already explained by a lower accessibility of catechols after particle shrinking and layer formation. In contrast, the preloaded PEC NP systems featured a low and short-term release, reaching saturation even after 10 h. Obviously, it is the state of the PEC NP (dispersion or coating) which is crucial for both the loading and release performance. In the end, the higher LC of preloaded PEC NP leads to higher residual contents and faster saturation reaching.

Analogous to the kinetic loading data, the Ritger, Korsmeyer, and Peppas model was used to represent the kinetic release data. The optimized parameters A_0_ characterizing the initial burst and B characterizing the diffusion type introduced above (loading) are summarized in Table 2. Concerning A_0_, no big difference between pre- and postloaded PEC NP was obtained for PEC 0.7 (around 20%), whereas for PEC 1.4 A_0_ was significantly lower (around 3%). Hence, for PEC 0.7 the initial burst release is similar, while for PEC 1.4, the initial burst is different for pre- compared to postloading. Presumably, the formation of a dense layer consisting of fused PEC 1.4 NP leads to this strong BZM retention upon preloading. Postloading of such a dense layer leads to quite low LC (see Figure 10) but obviously not to a reduced A_0_ in comparison to postloaded PEC 0.7, whereby the RC after 48 h is significantly different in the dependence of net charge. Furthermore, there is a significant difference between exponents B in dependence of the loading procedure of cationic PEC NP. The postloaded PEC 0.7 (B = 0.35) exhibits a more sustained release in comparison to the preloaded one (B = 0.09). This stronger retention is potentially based on the higher drug availability and has not so much to do with the shrinking of PEC NP during film formation. Presumably, a BZM molecule which is bound deep into the layer is able to release PEC coating using the same diffusion path as for the postloading process. This scenario is not imaginable for the preloading case. The layer morphology should be equal, but the accessibility of BZM-catechol ester versus free (unloaded) catechols is shifted due to a random distribution of diffusion pathways. Thus, it is crucial whether before or after layer formation the drug loading was performed. This trend was not observed for B of pre- and postloaded anionic PEC 1.4. Instead, the exponents are rather equal. Probably, the formation of the above-mentioned dense layer dominates the diffusion behavior of PEC 1.4 coatings, independent on the type of loading.

**Figure 16:**
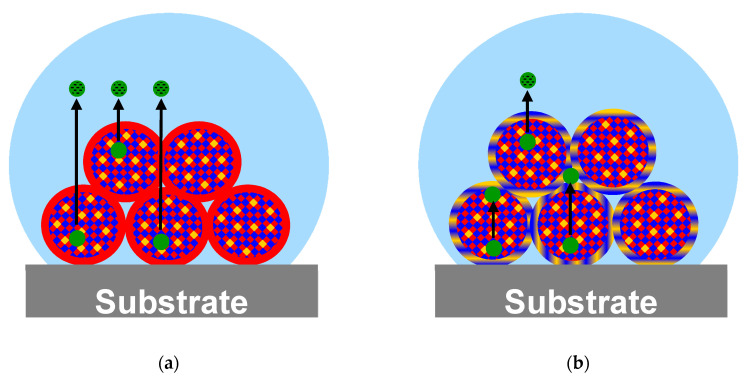
Illustrative presentation of PEC NP immobilized onto the BSM surface during BZM release from (**a**) positively charged PEC 0.7 and (**b**) negatively charged PEC 1.4. Schematically displayed is the less hindered diffusion of the drug through the PEC 0.7 coating in comparison to rebinding of BZM in the PDOPAC containing shell of PEC 1.4. Colors were chosen as follows: PLL: Red; PLG: Blue; PDOPAC: Yellow; covalently bound drug: Green; free drug: Green/black dashed; and release medium: Bright blue.

Cationic PEC 0.7 shows for each type of loading a higher BZM availability compared to anionic PEC 1.4 (see Figure 15). For loading, an analogous trend was obtained with respect to LC, such that the LC of PEC 1.4 was significantly lower than that of PEC 0.7 (see Table 1). Obviously, PDOPAC located in the shell of PEC NP has a barrier effect for both drug uptake and delivery schematically shown in Figure 16. The high catechol concentration in the shell probably leads to rebinding of the already released BZM (hydrolyzed BZM-PDOPAC ester). In cationic PEC 0.7, the PDOPAC distribution is more homogeneous and the drug is able to penetrate the shell consisting of polycation. Furthermore, it is possible that the shell of anionic PEC is denser, because of the low molecular weight of PDOPAC. Another explanation for the permanently higher RC of anionic PEC 1.4 is that the BZM which is mostly bound nearby the shell of NP is easier and faster to remove from the shell during the rinsing step. Loss of drug between loading and release would lead to an apparently higher RC.

Comparing the desorption behavior of postloaded PEC 0.7 at Ti40Nb with the same sample at germanium in Figure 13 we obtain a slightly accelerated release for the model substrate. The main reason could be the increased layer thickness of the PDOPAC/PEC at Ti40Nb in comparison to the coating at germanium, because the area for solution casting is roughly one third of that of germanium. Hence, the increase of layer thickness has two consequences: Hindering of BZM diffusion and slowing of water influx.

However, it is possible to adjust the release performance of PDOPAC/PEC NP coatings by varying the preparation parameters. The net charge, the type of loading as well as the layer thickness affect the drug release behavior.

##### Comparison to Charged Drugs and Influence of Salt (Ionic Strength)

**Figure 17:**
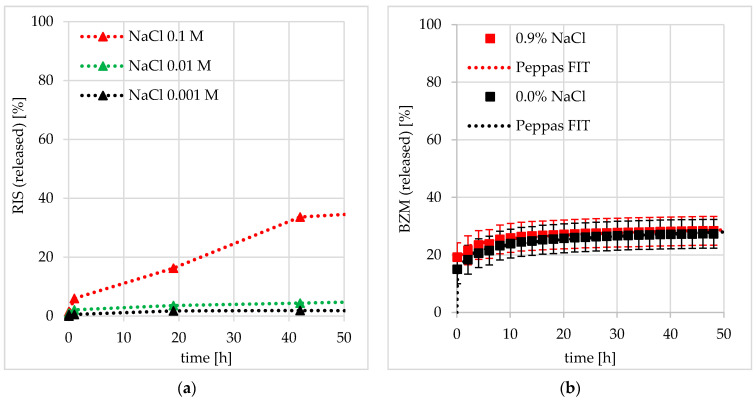
Drug release kinetics from cationic PEC 0.7 @ Ti40Nb in dependence of NaCl concentration in the release medium for (**a**) negatively charged risedronate (RIS) and (**b**) uncharged BZM.

In the former work [10], the influence of salt concentration in the release medium on the release kinetics of electrostatically bound drugs was studied. There, with the increasing NaCl content, the desorption of charged RIS was accelerated due to the decrease in Debye length. Herein, in contrast, the desorption of uncharged BZM is completely independent on the salt concentration, as shown in Figure 17. Obviously, ester hydrolysis as well as diffusion paths in the PEC coating cannot be affected by the ionic strength.

## 4. Conclusions

Novel catechol containing polyelectrolyte complex nanoparticle (PEC NP) coatings were fabricated based on PDOPAC, which bind temporarily to the boronic acid containing drug bortezomib and can release it in a sustained manner. In this study, we used an optimized PEC NP system based on refining the dispersions by (multiple) centrifugation and redispersion steps. Such refined PEC NP systems featured improved performance related to both higher loading and sustained release properties. Simultaneously, access to the drug content measurements in the supernatant by spectroscopic methods became possible. Furthermore, the adhesiveness to substrates such as bone substitute materials (BSM) of refined PEC NP is optimized due to the removal of excess polyelectrolytes by centrifugation. Additionally, the processing of PEC NP dispersions in buffer solutions at constant pH values, introduced in this study, reduced the risk of uncontrolled particle aggregation and precipitation. Thus, the preparation of colloidally-stable PEC NP dispersions with reproducible particle size is possible. In addition to these important colloidal properties, we addressed the loading and release of uncharged BZM into PDOPAC/PEC NP based on specific boronic acid–catechol interaction. For an effective loading and release of BZM the integration of chemo-reactive PDOPAC into PEC NP was mandatory, whereby the loading capacity (LC) strongly depended on the PDOPAC amount. Both the loading and the release performance of PDOPAC/PEC coatings were dependent on the net charge affecting the core/shell composition of PEC NP and the type of loading (pre- and postloading). Cationic PEC 0.7 showed the more effective BZM loading and the more effective as well as sustained release compared to PEC 1.4. This could be explained by the polyelectrolyte distribution related to the model of charged compensated core and charge uncompensated shell structure of PEC NP. Furthermore, the loading and release performance of the coating was independent on the substrate material (CPC, Ti40Nb, Ge), from which the unique character of drug delivery systems based on PEC NP can be concluded. For the uncharged BZM, drug loading and release was independent, while for the negatively charged risedronate (RIS), the loading and release was strongly dependent on ionic strength, which significantly classifies as electrostatic in comparison to the chemical binding of RIS and BZM, respectively. We could demonstrate that the newly developed DDS might be beneficial in the context of treating multiple myeloma related bone lesions.

## Figures and Tables

**Table 1:** Kinetic parameters A_0_ and B of BZM loading at PDOPAC/PEC NP at c_BZM_ = 0.25 mM.

Kinetic Parameter	Preloading		Postloading	
	PEC 0.7	PEC 1.4	PEC 0.7	PEC 1.4
Amplitude A_0_ [mM]	0.02212 ± 0.00020	0.01384 ± 0.00067	0.01107 ± 0.00059	0.00683 ± 0.00039
Exponent B [-]	0.06702 ± 0.00508	0.13697 ± 0.02501	0.18037 ± 0.02717	0.18923 ± 0.02884

**Table 2:** Kinetic parameters A_0_ und B of BZM release at PDOPAC/PEC NP.

Kinetic Parameter	Preloading		Postloading	
	PEC 0.7	PEC 1.4	PEC 0.7	PEC 1.4
Amplitude A_0_ [mM]	20.1102 ± 2.3925	3.3194 ± 0.4150	23.3205 ± 0.5028	21.5088 ± 2.4807
Exponent B [-]	0.0884 ± 0.0260	0.1753 ± 0.0179	0.3521 ± 0.0107	0.1620 ± 0.0035

## Supplementary Materials

The following are available online at https://www.mdpi.com/1999-4923/12/9/799/s1. Figure S1: ATR-FTIR spectrum of the polymerisate PDOPAC in comparison to that of the monomer DOPAC, Figure S2: ATR-FTIR spectrum of PDOPAC/BZM in comparison to spectra of pure BZM and PDOPAC at pH 7.4, Figure S3: The evolution of count rate of PEC 0.7 in dispersion measured by DLS, Figure S4: The evolution of PDI of PEC 0.7 in dispersion measured by DLS, Figure S5: The evolution of hydrodynamic radii of PEC 1.4 in dispersion measured by DLS, Figure S6: The evolution of count rate of PEC 1.4 in dispersion measured by DLS, Figure S7: The evolution of PDI of PEC 1.4 in dispersion measured by DLS, Figure S8: Modified isotherm for the calculation of specific bound BZM (Subtraction of physisorbed drug), Table S1: Parameters of the Langmuir fit on BZM sorption at various PEC samples, Table S2: Zeta Potential values of PEC 0.7 PLG:PDOPAC 3:1 and PEC 1.4 PLG:PDOPAC 3:1 at pH = 7.4 (HEPES).
