# Supplementary material for: Catechol Containing Polyelectrolyte Complex Nanoparticles as Local Drug Delivery System for Bortezomib at Bone Substitute Materials
Source: Pharmaceutics. 2020 Aug 24;12(9):799. doi: 10.3390/pharmaceutics12090799 (PMC7557399; doi:10.3390/pharmaceutics12090799)
Supplement: Supplementary file 1 [file pharmaceutics-12-00799-s001.pdf]

pharmaceutics

Supplementary Materials: Catechol containing polyelectrolyte complex nanoparticles as local drug delivery system for bortezomib at bone substitute materials

David Vehlow, Jeremy P. H. Wong, Birgit Urban, Janek Weißpflog, Annett Gebert, Michael Gelinsky, Manfred Stamm and Martin Müller

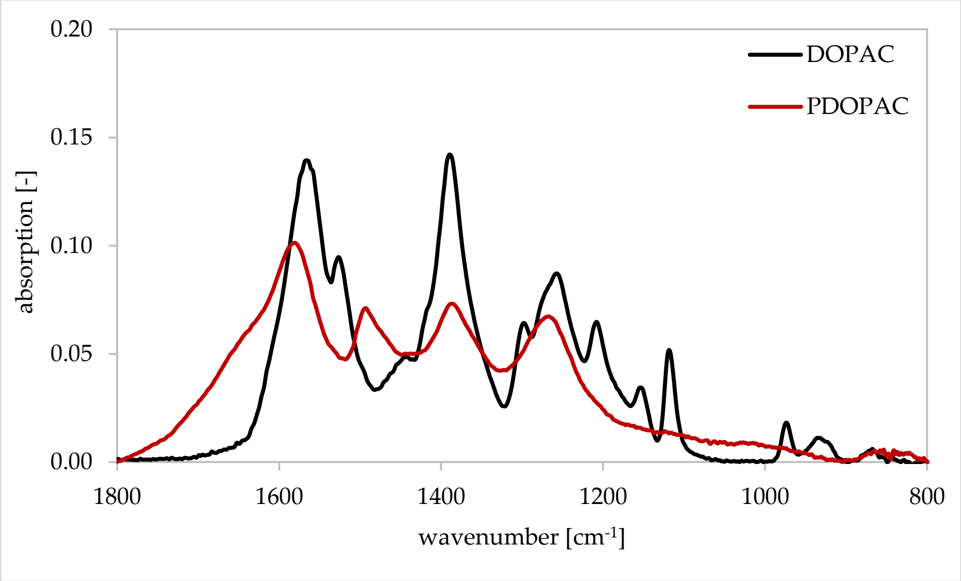

Figure S1. ATR-FTIR spectrum of the polymerisate PDOPAC in comparison to that of the monomer DOPAC.

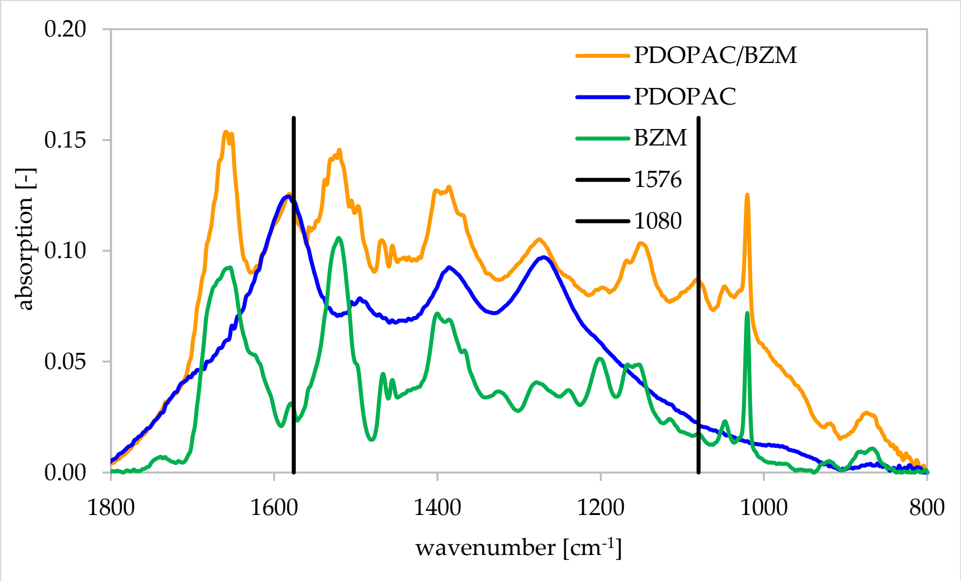

Figure S2. ATR-FTIR spectrum of PDOPAC/BZM in comparison to spectra of pure BZM and PDOPAC at pH 7.4.

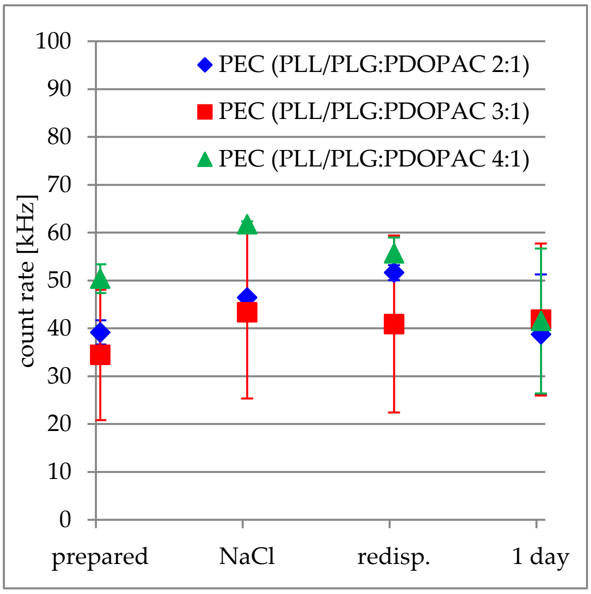

Figure S3. The evolution of count rate of PEC 0.7 in dispersion measured by DLS**.**

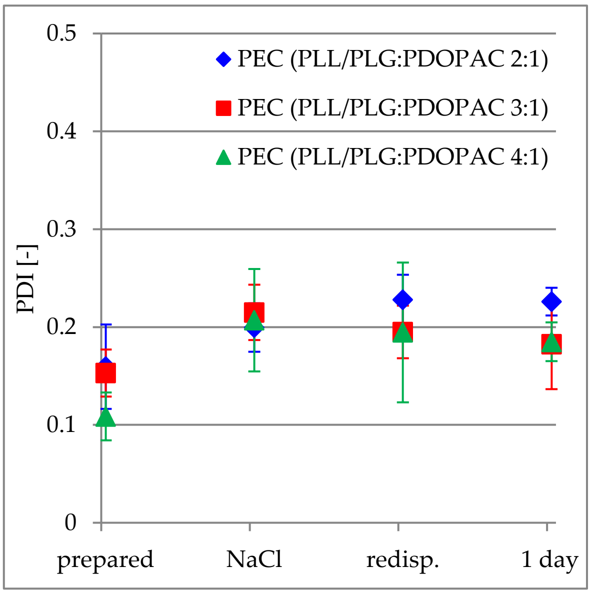

Figure S4. The evolution of PDI of PEC 0.7 in dispersion measured by DLS.

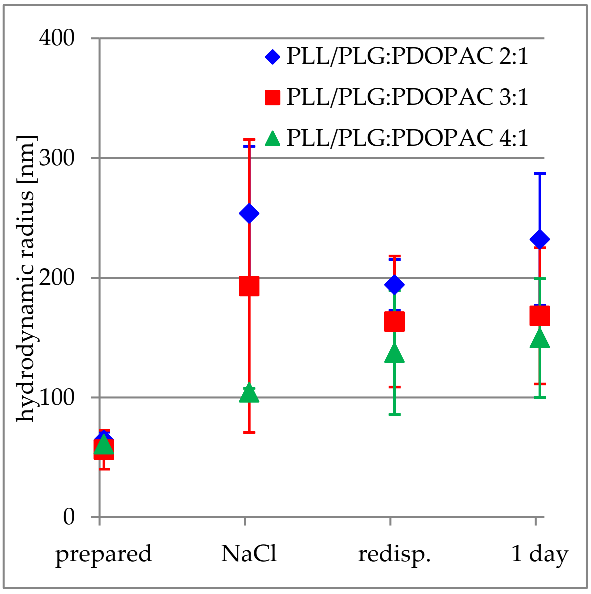

Figure S5. The evolution of hydrodynamic radii of PEC 1.4 in dispersion measured by DLS.

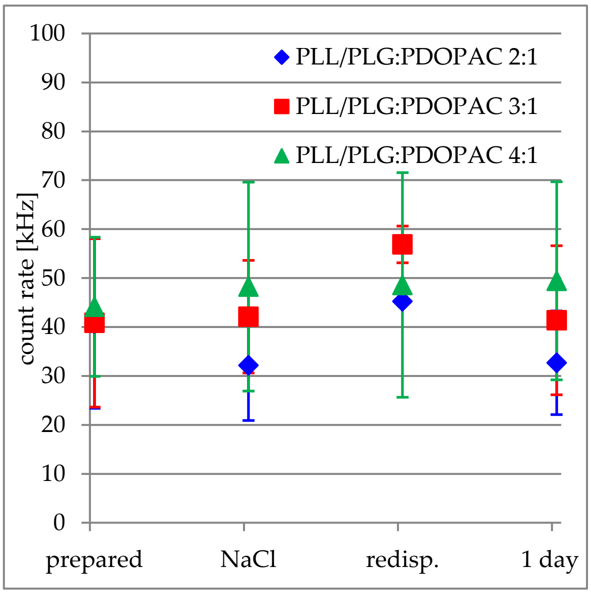

Figure S6. The evolution of count rate of PEC 1.4 in dispersion measured by DLS.

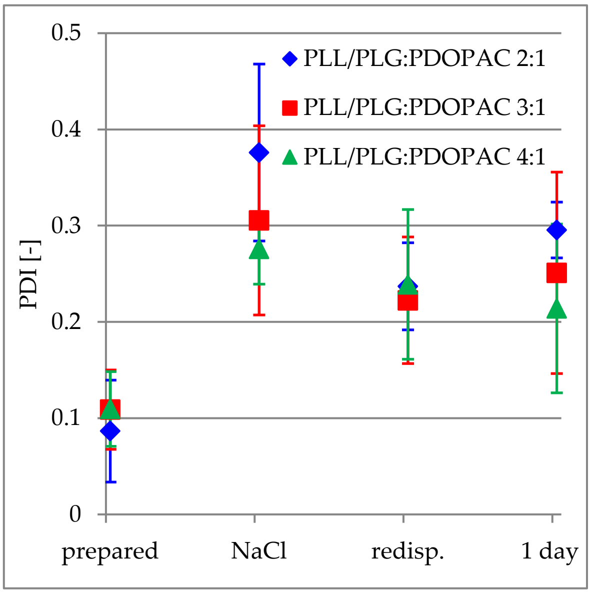

Figure S7. The evolution of PDI of PEC 1.4 in dispersion measured by DLS.

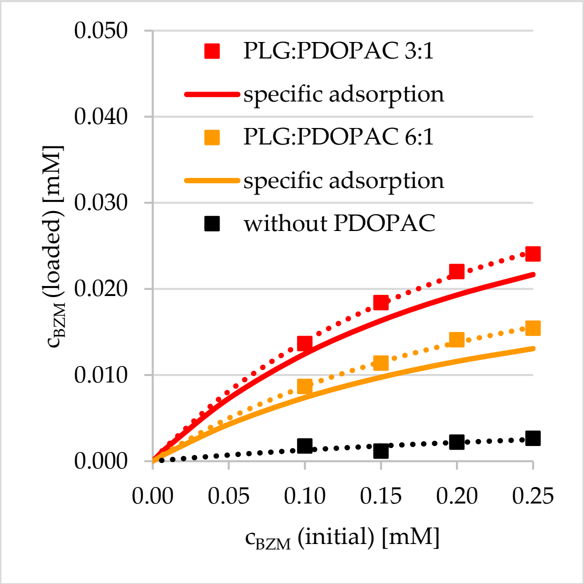

Figure S8. Modified isotherm for the calculation of specific bound BZM (Subtraction of physisorbed drug).

Table S1. Parameters of the Langmuir fit on BZM sorption at various PEC samples.

Sample	c_{max} [mM]	K_L [-]	R^2 [-]
**Concentration dependency**			
PEC 0.7 PLG without PDOPAC-preloading	0.00678	2.36726	0.49795
PEC 0.7 PLG:PDOPAC 3:1-preloading	0.05167	3.79834	0.98561
PEC 0.7 PLG:PDOPAC 6:1-preloading	0.03316	3.55104	0.99450
PEC 0.7 PLG:PDOPAC 3:1-postloading	0.03195	3.50505	0.99738
**Volume dependency**			
PEC 0.7 PLG without PDOPAC-preloading	0.02186	0.21821	0.95846
PEC 0.7 PLG:PDOPAC 3:1-preloading	0.08810	0.16197	0.99412

Table S2. Zeta Potential values of PEC 0.7 PLG:PDOPAC 3:1 and PEC 1.4 PLG:PDOPAC 3:1 at pH = 7.4 (HEPES).

Sample	Zeta Potential [mV]
PEC 0.7 PLG:PDOPAC 3:1	+29.4 ± 0.9
PEC 1.4 PLG:PDOPAC 3:1	−32.0 ± 0.3
